# Serosurvey of *Rickettsia* spp. and *Ehrlichia canis* in Dogs from Shelters in Sicily (Southern Italy)

**DOI:** 10.3390/pathogens13121119

**Published:** 2024-12-18

**Authors:** Santina Di Bella, Delia Gambino, Davide Pepe, Antonino Gentile, Valeria Blanda, Antonio Valenti, Francesco Santangelo, Antonino Ballatore, Giuseppe Spina, Giuseppe Barbaccia, Vincenza Cannella, Giovanni Cassata, Annalisa Guercio

**Affiliations:** 1 Centro di Referenza Nazionale per Anaplasma, Babesia Rickettsia, e Theileria (C.R.A.Ba.R.T.), Istituto Zooprofilattico Sperimentale della Sicilia “A. Mirri”, 90129 Palermo, Italy; santina.dibella@izssicilia.it (S.D.B.);; 2 Area Territoriale Palermo, Istituto Zooprofilattico Sperimentale della Sicilia “A. Mirri”, 90129 Palermo, Italy; delia.gambino@izssicilia.it (D.G.); giuseppe.barbaccia@izssicilia.it (G.B.);; 3 Dipartimento di Prevenzione Veterinario UOS Presidi di Igiene Urbana Veterinaria (Canile), Azienda Sanitaria Provinciale di Palermo, 90123 Palermo, Italy; 4 Rifugio Sanitario Municipale di Mazara del Vallo, Azienda Sanitaria Provinciale di Trapani, 91026 Mazara del Vallo, Italy

**Keywords:** *Rickettsia* spp., *Ehrlichia canis*, dogs, shelter, One Health

## Abstract

Vector-borne diseases represent a serious threat to human and animal health, especially where environmental conditions favor pathogen-carrying vectors. Dogs serve as natural hosts for two tick-borne pathogens: *Ehrlichia canis*, which causes canine monocytic ehrlichiosis, and spotted fever group (SFG) *Rickettsia* spp., a zoonotic threat in the Mediterranean region. *Rhipicephalus sanguineus* is the primary vector for these pathogens. Shelter dogs, due to increased exposure to ticks and confined living conditions, facilitate the spread of vector-borne pathogens, raising the risk of zoonotic transmission. This study conducted a serological survey of 1287 dogs from two shelters, assessing exposure to *Rickettsia* spp. and *E. canis* and examining the influence of demographic and environmental factors. Seroprevalence rates were 41.8% for *Rickettsia* spp. and 24.5% for *E. canis*, with 14% of dogs positive for both pathogens. No significant association was found with sex or breed. A higher seroprevalence was observed in dogs older than 12 months and in those from the shelter on the Mediterranean coast compared to those from the Tyrrhenian coast, likely due to climatic differences. The study highlights the role of climate in disease spread and the need for public health interventions, supporting One Health initiatives to prevent zoonotic disease transmission.

## 1. Introduction

Tick-borne diseases, caused by parasites, viruses, and bacteria, pose a significant risk to both human and animal health, particularly in areas where environmental conditions favor the proliferation of vectors [1,2].

The Mediterranean climate, with its hot-arid summers and mild-humid winters, is particularly favorable for the survival and spread of several tick species, thereby influencing the geographical distribution of tick-borne diseases in the region [3].

The brown dog tick, *Rhipicephalus sanguineus*, also known as the kennel tick, is the primary vector in the Mediterranean area [4]. Its prevalence and geographic spread are closely related to the Mediterranean climate, which supports its biological lifecycle and interaction with host species [5].

*Rhipicephalus sanguineus* acts as a relevant vector for several pathogens, including *Rickettsia* spp. and *Ehrlichia canis*, both of which belong to the order *Rickettsiales* [4,6]. *Rickettsia* and *Ehrlichia* are obligate intracellular Gram-negative bacteria that undergo part of their life cycles within ticks, though they differ in their transmission dynamics [2]. *Rickettsia* can be transmitted both transovarially and transstadially within tick populations, ensuring continuous pathogen circulation across generations and developmental stages [2]. *Rhipicephalus sanguineus* represents a pivotal vector in the *Rickettsia* life cycle, with evidence suggesting it may serve as a reservoir host for the bacteria [5]. In contrast, *Ehrlichia* is exclusively transmitted transstadially, with the pathogen persisting through successive tick life stages but not transmitted vertically from female ticks to their offspring [7].

*Ehrlichia canis*, belonging to the family *Anaplasmataceae*, is the main causative pathogen of canine monocytotropic ehrlichiosis (CME), a serious and potentially lethal immunosuppressive disease affecting dogs [8]. Clinical manifestations of *E. canis* infection range from subclinical to fatal, and they include fever, lethargy, weight loss, and hematological abnormalities [9]. In Europe, *E. canis* is the sole *Ehrlichia* species found in canine hosts, and all European countries bordering the Mediterranean Sea are considered endemic for this pathogen [1,6]. Asymptomatic or paucisymptomatic infections have occasionally been reported in humans in various regions, including the United States [10,11], Venezuela [12], and Costa Rica [13].

*Rickettsia conorii* is responsible for causing Mediterranean spotted fever (MSF), a disease predominantly found in the Mediterranean region, southern Europe, North Africa, sub-Saharan Africa, and the Middle East [2,14]. MSF is endemic to these regions and typically starts suddenly in humans with fever, influenza-like symptoms, a necrotic eschar at the site of the tick bite, and a maculopapular rash [15]. In severe instances, the condition can progress to serious neurological complications and multi-organ involvement [16].

In Italy, almost all human cases of rickettsial diseases are due to Mediterranean spotted fever (MSF), caused by *R. conorii* and spread by *Rh. sanguineus*. The highest incidence is seen in individuals from Sicily, Sardinia, and southern Italy, with a mortality rate of less than 3%. Nevertheless, numerous other *Rickettsia* species or subspecies have been recently identified in humans, arthropod vectors, and animals [17]. Furthermore, several rickettsia species such as *R. helvetica*, *R. massiliae*, *R. rhipicephali*, *R. monacensis*, *R. slovaca*, *Candidatus R. barbariae*, *R. raoultii*, have been detected in *Rh. sanguineus* [17].

The life cycle of *Rh. sanguineus* and the transmission of pathogens it carries can be influenced by climatic factors. Moderate to warm temperatures accelerate the tick’s development, while extreme temperatures can hinder activity [6,14]. High relative humidity enhances tick survival and transmission, whereas low humidity impedes their development [18]. Previous studies reported that in addition to climatic variables, land use, vegetation, and annual precipitation were also associated with the distribution of this tick species [19,20]. Wind reduces the chance of tick attachment to hosts [3], and seasonality plays a role, with tick activity peaking during warm, humid months and decreasing in colder periods [16]. These climatic variables collectively shape the transmission patterns of *E. canis* and *Rickettsia* spp., influencing their geographical distribution and seasonal prevalence.

Shelter dogs can serve as reservoirs for ticks due to the limited spaces in which they coexist and the overcrowded conditions, potentially contributing to the transmission of ticks and vector-borne infections to other animals [1,6,21,22]. Furthermore, since these dogs often form close bonds with humans or are adopted, the potential for zoonotic transmission represents a significant public health concern [22]. Previous studies have highlighted a high prevalence of *E. canis* and *R. conorii* infections in shelter dogs across the Mediterranean region [23,24]. The shelter dog population plays a pivotal role within the One Health framework, which emphasizes the interrelationship of human, animal, and environmental health [25]. Therefore, conducting sero-epidemiological surveys and subsequent tick control and treatment protocols in shelter dogs is vital not only for their welfare but also to reduce the risk of pathogen transmission to humans and to safeguard public health.

In the context of ongoing monitoring of the circulation of *Rickettsia* spp. and *E. canis* in Sicily (southern Italy), this study aimed to perform a serological survey of these two pathogens in dogs from two animal shelters located in the western part of the region. The study specifically focused on evaluating potential associations between demographic factors (such as sex, breed, and age) and the area of origin of the dogs that exhibited higher seroprevalence rates. The results will provide important updates on the epidemiology of these pathogens in western Sicily, contributing to evidence-based veterinary practices and public health strategies within the One Health framework.

## 2. Materials and Methods

### 2.1. Study Area

**Figure 1:**
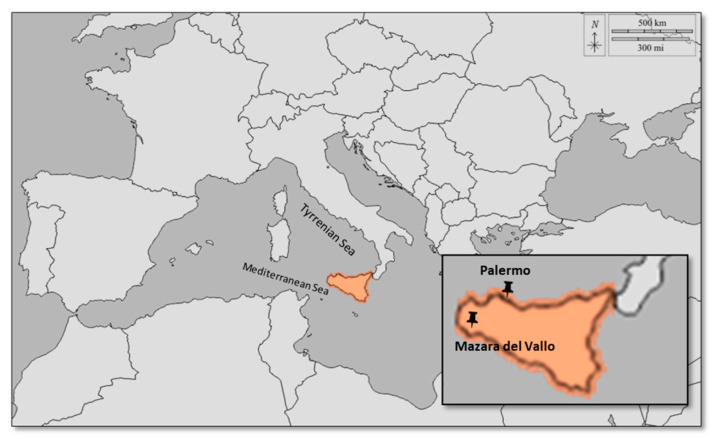
Geographical distribution of the study areas, with Mazara del Vallo located on the Mediterranean coast and Palermo on the Tyrrhenian coast of Sicily.

The study was conducted in two public shelters: one located in Mazara del Vallo (Trapani), (Lon 12.61328; Lat 37.66951) on the southwestern Mediterranean coast of Sicily, and one in Palermo (Lon 13.2237; Lat 38.06366) on the northwestern coast of Sicily on the Tyrrhenian side of the island (Figure 1).

Data on annual temperature, daily total precipitation, daily maximum relative humidity, and daily wind speed, collected through the Servizio Informativo Agrometeorologico Siciliano (http://www.sias.regione.sicilia.it/ accessed on 21 September 2024) for the years 2022 and 2023, indicate that these two Sicilian cities exhibit climatic differences due to their geographical locations and environmental characteristics relevant to vector-borne diseases. Specifically, Palermo is characterized by higher average annual temperatures and greater estimated daily total precipitation compared to Mazara del Vallo. In contrast, Mazara del Vallo records higher average daily wind speeds and greater average daily maximum relative humidity than Palermo (Table 1).

The two shelters adhere to current regulations governing animals and their welfare. Specifically, they administer vaccinations and anti-parasitic prophylaxis upon the animals’ entry and exit and periodically conduct rodent control and anti-parasitic treatments within the facility.

### 2.2. Sample Collection

The animals appeared to be in good health at the time of admission to the shelter; however, their medical history was unavailable as they were all stray dogs. Information regarding each animal’s sex, age, breed, and shelter location was recorded. Whole blood and ethylenediaminetetraacetic acid (EDTA) blood samples were collected from dogs at the two shelters as part of routine testing. Whole blood samples were centrifuged at 1500× *g* for 15 min, after which the serum was isolated from the clot, and the samples were either analyzed promptly or preserved at −20 °C for later use. DNA was extracted from the EDTA blood samples and stored at −80 °C until use for biomolecular analysis.

### 2.3. Serological Tests

Antibodies to *Rickettsia* spp. and *E. canis* were detected by the commercial tests *Ehrlichia canis* IgG IFA kit and the Canine *Rickettsia conorii* IgG IFA kit (both from Fuller Laboratories, Fullerton, CA, USA) following manufacturer’s instructions. Sera were diluted to 1:50 for *Ehrlichia* and 1:64 for *Rickettsia* with 1X phosphate-buffered saline (PBS), as recommended by the manufacturers. The results were observed under standard fluorescence microscopy, where a positive reaction was indicated by bright staining of short pleomorphic rod forms and chains of small coccobacilli, with specific staining visible within the cytoplasm of cells. Positive and negative controls were included in the kit. Sera with titers ≥ 1:50 for *E. canis* and ≥1:64 for *Rickettsia* spp. were considered positive.

### 2.4. Biomolecular Analysis

DNA was extracted from EDTA blood samples using the DNeasy blood and tissue kit (Qiagen, Germany) following the manufacturer’s protocol.

The extracted DNA was analyzed using PCR, amplifying the outer membrane protein A (*OmpA*) [26] and B (*OmpB*) [27] genes, as well as the citrate synthase (*gltA*) [28] gene for the detection of *Rickettsia* spp. DNA, and the *16S-rRNA* gene for detecting *E. canis* DNA [29,30] (Table 2).

The PCR assays were conducted in a total volume of 50 µL, employing GoTaq G2 DNA Polymerase (Promega Italia s.r.l., Milan, Italy), with 5 µL of each DNA sample. Positive and negative controls were included in each amplification assay. The amplicons were visualized by electrophoresis on a 2% agarose gel. To confirm the positive PCR results, the amplicons were quantified and subsequently sequenced by Macrogen Inc. (Macrogen Europe, Amsterdam, The Netherlands).

The sequences generated were analyzed with BioEdit software version 7.2.5 (Tom Hall, Ibis Biosciences, Carlsbad, CA, USA) and compared to reference strains in the GenBank database using the Basic Local Alignment Search Tool (BLAST) to assess nucleotide sequence similarity.

### 2.5. Statistical Analysis

A binomial test was applied to compute the confidence intervals (CI) for the proportions, using a 95% confidence level. The chi-square test and Fisher’s exact test were employed to compare the proportions of positivity associated with categorical dependent variables. A *p*-value less than 0.05 was deemed to indicate statistical significance.

## 3. Results

### 3.1. Study Population

A total of 1287 dogs were examined: 719 (55.8%) were male and 568 (44.1%) were female; 384 (29.8%) were younger than one year and 903 (70.1%) were older than one year; 1091 (84.7%) were sampled from the Palermo shelter and 196 (15.2%) from the Mazara del Vallo shelter; 1113 (86.4%) were mixed-breed and 174 (16.5%) were purebred.

### 3.2. Serological Results

The study revealed an overall seroprevalence of 41.8% (*n* = 538) (95% CI: 39.4–44.4%) for *Rickettsia* spp. and 24.5% (n = 316) (95% CI: 22.2–26.9%) for *E. canis* among the sampled dogs. A total of 179 dogs (13.9%; 95% CI: 12–15.7%) tested positive for both pathogens.

#### 3.2.1. *Rickettsia* spp.

A statistically significant difference was observed with respect to age (*p* < 0.0001); specifically, dogs older than one year showed a prevalence of 45.5% (95% CI: 42.2–48.8%), compared to 33.1% (95% CI: 28.4–37.8%) observed in dogs younger than one year. With regard to the shelter of origin, dogs from Mazara del Vallo exhibited a higher positivity percentage (56.1%; 95% CI: 49.2–63%) compared to those from Palermo (39.3%; 95% CI: 36.4–42.2%), with the difference being statistically significant (*p* < 0.0001). No significant correlation was observed between sex or breed and *Rickettsia* spp. prevalence (Table 3).

#### 3.2.2. *Ehrlichia canis*

With regard to the shelter of origin, dogs from Mazara del Vallo showed a higher positivity percentage (31.1%; 95% CI: 24.6–37.6%) compared to those from Palermo (23.4%; 95% CI: 20.8–58.8%), with the difference being statistically significant (*p* = 0.021) (Table 3). No statistically significant association was observed for *E. canis* in relation to sex, age, or breed (Table 3).

#### 3.2.3. Coinfections

When comparing the results between different age groups, statistically significant differences were observed regarding coinfections (*p* = 0.006). Specifically, prevalence was higher in dogs older than 12 months (15.6%; 95% CI:13.2–18%) compared to those younger than 12 months (9.8%; 95% CI: 6.9–12.8%). Regarding origin, the lower prevalence of coinfections was observed in the shelter of Palermo (13%; 95% CI: 11–15%), while the higher prevalence was recorded in the shelter of Mazara del Vallo (18.8%; 95% CI: 13.5–24.3%), with a statistically significant difference (*p* = 0.030). No statistically significant association was observed for coinfections in relation to sex and breed (Table 3).

### 3.3. Biomolecular Results

Biomolecular analyses for *Rickettsia* spp. and *E. canis* were conducted on EDTA blood samples from approximately 20% of the animals involved in the study (260 dogs), randomly selected from the two shelters.

Two dogs, one of which was seropositive and the other seronegative, both tested positive by PCR for *E. canis* (0.8%), while no dogs tested positive for *Rickettsia* spp.

The ticks were not available for analysis because the dogs were treated with acaricides when they entered the shelter to control ectoparasites.

## 4. Discussion

This study provides important insights into the prevalence and distribution of *E. canis* and *Rickettsia* spp. among shelter dogs from two Sicilian cities: Palermo and Mazara del Vallo. The overall observed seroprevalence rates, 41.8% for *Rickettsia* spp. and 24.5% for *E. canis*, underscore the significant exposure of these dogs to vector-borne pathogens, likely due to the widespread circulation of *Rh. sanguineus* throughout the Mediterranean basin [31], where environmental conditions promote tick proliferation. These findings are consistent with prior research indicating that the warm temperatures and seasonal humidity typical of the Mediterranean climate enhance tick activity and distribution [23,32].

The high serological positivity detected by immunofluorescence for *R. conorii* is likely not entirely correlated with this microorganism, but also with other spotted fever group (SFG) rickettsiae present in the area, such as *R. helvetica*, *R. massiliae*, *R. slovaca*, *R. monacensis*, *R. aeschlimannii*, *R. raoultii*, *R. africae*, and others [33,34,35]. Indeed, the gold standard indirect fluorescent antibody (IFA) test does not distinguish infections caused by different SFG rickettsiae due to cross-reactivity [36,37].

*Ehrlichia canis* shares antigenic characteristics with the pathogenic canine species *E. ewingii* and *E. chaffeensis*, but the lack of detailed information about the dogs’ history introduces uncertainty in diagnostic interpretation. While cross-reactivity with other *Ehrlichia* species remains a theoretical concern, the predominance of *E. canis* as the sole documented *Ehrlichia* species in European canine populations suggests that such scenarios are improbable [38,39].

No significant associations were identified between sex or breed and the prevalence of either pathogen. Studies investigating the correlation between sex and the prevalence of *Rickettsia* spp. and *E. canis* in dogs have not yielded conclusive results. Some studies have found no significant differences between males and females [40,41], while other research indicates that males may be more susceptible, likely due to higher outdoor activity and exploratory behavior [42]. Conversely, Alonso et al., 2024 reported higher seroprevalence for both pathogens in female dogs [22].

Regarding the correlation between breed and *Rickettsia* spp. and *E. canis* prevalence in dogs, there is limited specific evidence [43]. However, certain breeds may have a predisposition to particular infectious diseases, influenced by genetic and environmental factors. Some specific breeds, such as Siberian Huskies and German Shepherds, may exhibit higher prevalence rates for *E. canis* [44,45,46], although this correlation can vary based on regional and local conditions.

Regarding age, dogs older than 12 months showed a higher incidence of *Rickettsia* spp. infections and a greater rate of coinfections compared to younger dogs. This may reflect the cumulative risk of exposure to *Rickettsia* spp. as the dog ages, rather than increased age-related susceptibility [47]. Additionally, the likelihood of infection from tick larvae further raises the risk of *Rickettsia* transmission. Older dogs may also have a weakened immune system, which can make them more vulnerable to both primary and secondary infections. In contrast, no significant age-related differences were observed for *E. canis*, suggesting that factors such as individual immunity or repeated pathogen exposure may play a more critical role in shaping infection dynamics for this particular pathogen [8].

Statistically significant differences were observed in positivity rates for *E. canis*, *Rickettsia* spp. and their coinfections based on the geographical origin of the dogs. In particular, in the Mediterranean coastal area of western Sicily, higher infection rates may be associated with environmental factors such as lower daily precipitation, higher average daily maximum relative humidity, and milder temperatures [48]. In contrast, the climatic conditions of the Tyrrhenian coastal area could impact tick distribution, leading to lower infection rates in dogs. Spatial and seasonal variability in climatic factors, even within regional contexts, can differentially affect the various life stages of ticks, as well as the dynamics between ticks and their hosts [19,31,49,50].

Identifying predisposing factors for tick-borne pathogen infections in free-ranging animals presents significant challenges, complicating the precise assessment of the role of climate. However, as climate change accelerates, its influence on the geographical distribution of vector-borne diseases becomes increasingly clear [51]. Abiotic factors such as temperature and relative humidity are crucial for the survival of *Rh. sanguineus* [4,31]. Studies have shown that *this species* can survive for longer periods without a blood meal compared to other tick species, likely due to its reduced water loss rate, which enhances its ability to withstand environmental stressors such as drought [52]. Vegetation type, relative humidity, and the length of the rainy season are key determinants influencing the dispersal and survival of *Rhipicephalus* ticks. Understanding the interaction between these biotic and abiotic factors and the questing behavior of ticks is essential for estimating both the spatial and temporal distribution of ticks and, consequently, the risk of tick-borne diseases [53].

This study also found an overall seroprevalence of 14% for coinfections. Coinfections may result from multiple scenarios: simultaneous exposure to different tick species, transmission of different pathogens through a single tick species, or interactions between geographically close disease vectors. These concurrent infections pose significant diagnostic challenges, potentially increasing disease progression and treatment complexity [2]. Although in this study the ticks were not collected, as the dogs had been treated with acaricides before entering the shelter, the presence in the area of *Rh. sanguineus*, known to be the main vector of both *E. canis* and *R. conorii*, cannot be excluded [17,54]. However, although less frequently, other *Rickettsia* species have been detected in various tick species collected from dogs in Italy, such as *Hyalomma marginatum*, *Ixodes ricinus*, *Dermacentor marginatus*, and *Hyalomma sulcata* [17].

Blood samples from approximately 20% of the dogs involved in the study were analyzed by PCR, without the intention of conducting a molecular surveillance study. All the dogs tested negative for *Rickettsia* spp. PCR does not always detect *Rickettsia* in the blood of infected subjects, as the bacteremic phase is of short duration. Shelter dogs may serve as sentinels for zoonotic pathogens such as *Rickettsia* spp., indicating the potential risk these diseases pose to other animals and humans [55].

Despite all dogs appearing to be in good health, two dogs tested positive for *Ehrlichia*, one of which was seronegative and the other seropositive. The difference between the PCR and serology results may be due to the dogs’ varying infection states. The seronegative but PCR-positive dog was likely in an early stage of *E. canis* infection, which can be detected by PCR in the blood [56]. Since this study only screened for IgG antibodies, which are not detected until 2–3 weeks post-infection [57], it is difficult to determine whether this represents the acute phase of the infection. The other dog, which tested positive in both serology and PCR, may be experiencing either an acute *E. canis* infection, a persistent chronic infection, or a subclinical infection, with bacteria intermittently circulating in the peripheral blood. Dogs act as a reservoir for *E. canis* as asymptomatic infection can persist for years, with the pathogen remaining present in chronically infected animals [55].

The fact that most of the seropositive dogs were PCR-negative may suggest past infections, subclinical infections, or chronic infections without active disease. The high seroprevalence of *E. canis* and *Rickettsia* spp. among shelter dogs underscores the urgent need for enhanced tick control measures within shelter environments. The confined and often stressful conditions in shelters can exacerbate tick infestations, creating an ideal environment for the spread of vector-borne diseases [58]. To mitigate this risk, shelters should implement regular tick prevention protocols and health screenings for dogs. Furthermore, educating shelter staff and dog owners about the importance of tick control and the health risks posed by these pathogens is crucial for reducing the potential health risks to both animals and humans.

This study provides updated data on the region concerning the spread of these pathogens, contributing to their assessment within a broader One Health perspective by emphasizing the interconnection between human, animal, and environmental health [59]. Therefore, integrated management strategies that address health risks across species are essential for controlling the spread of these vector-borne diseases.

## 5. Conclusions

In conclusion, this study provides valuable insights into the high rate of seroprevalence of *Rickettsia* spp. and *E. canis* in shelter dogs in Sicily, contributing to our understanding of the epidemiology of these pathogens in the region. The findings underscore the importance of public health interventions aimed at controlling vector-borne diseases, aiding in the identification and mitigation of infection hotspots to prevent disease transmission to both animals and humans, thereby promoting One Health initiatives and developing targeted control strategies.

## Figures and Tables

**Table 1:** Climatic data recorded in Palermo and Mazara del Vallo for the years 2022 and 2023 (http://www.sias.regione.sicilia.it/ accessed on 21 September 2024).

Years	Locations	Average Temperature (°C)	Estimated Daily Total Precipitation (mm)	Average Daily Maximum Relative Humidity (%)	Average Daily Wind Speed (m/s)
2022	Palermo	19.3	582.8	79.9	1.5
	Mazzara del Vallo	17.9	350.6	90.7	3
2023	Palermo	19.3	621.4	79.9	1.5
	Mazzara del Vallo	17.6	384.8	89.4	2.8

**Table 2:** PCR performed for the amplification of different *Rickettsia* spp. molecular targets (*OmpA*, *OmpB*, and *gltA*) and *Ehrlichia canis* (*16S-rRNA*).

Pathogen	Primers	Method	Target Gene	Reference
*Rickettsia* spp.	Rr190.70p 5′-ATGGCGAATATTTCTCCAAAA-3′ Rr190.701n 5′-GTTCCGTTAATGGCAGCATCT-3′ Rr190.602n 5′-AGTGCAGCATTCGCTCCCCCT-3′	Semi-nested PCR	*OmpA*	[26]
*Rickettsia* spp.	rompB OF 5′-GTAACCGGAAGTAATCGTTTCGTAA-3′ rompB OR 5′-GCTTTATAACCAGCTAAACCACC-3′ rompB SFG IF 5′-GTTTAATACGTGCTGCTAACCAA-3′ rompB SFG IR 5′-GGTTTGGCCCATATACCATAAG-3′	Nested PCR	*OmpB*	[27]
*Rickettsia* spp.	RpCS.877p 5′-GGGGGCCTGCTCACGGCGG-3′ RpCS.1258n 5′-ATTGCAAAAAGTACAGTGAACA-3′	PCR	*Citrate synthase*	[28]
*Ehrlichia canis*	ECC 5′-AGAACGAACGCTGGCGGCAAGCC-3′ ECB 5′-CGTATTACCGCGGCTGCTGGCA-3′ CANIS 5′-CAATTATTTATAGCCTCTGGCTATAGGA-3′ HE3 5′-TATAGGTACCGTCATTATCTTCCCTAT-3′	Nested PCR	*16S-rRNA*	[29,30]

**Table 3:** Seroprevalence of *Ehrlichia canis*, *Rickettsia* spp., and coinfections by demographic characteristics of the dogs included in the study.

Category	*Rickettsia* spp. Positive/Total (%)	95% CI	*E. canis* Positive/Total (%)	95% CI	Co-Infection Positive/Total (%)	95% CI
Sex	*p* = 0.772		*p* = 0.172		*p* = 0.244	
Male	303/719 (42.1%)	38.5–45.7%	187/719 (26%)	22.8–29.2%	104/719 (15.5%)	11.9–17%
Female	235/568 (41.3%)	37.3–45.4%	129/568 (22.7%)	19.2–26.1%	75/568 (13.2%)	10.4–16%
Age	*p* < 0.0001 *		*p* = 0.137		*p* = 0.006 *	
<1 year	127/384 (33.1%)	28.4–37.8%	84/384 (21.8%)	17.7–26%	38/384 (9.8%)	6.9–12.8%
>1 year	411/903 (45.5%)	42.2–48.8%	232/903 (25.7%)	22.8–28.5%	141/903 (15.6%)	13.2–18%
Shelter of origin	*p* < 0.0001 *		*p* = 0.021 *		*p* = 0.030 *	
Palermo	428/1091 (39.3%)	36.4–42.2%	255/1091 (23.4%)	20.8–58.8%	142/1091 (13%)	11–15%
Mazara del Vallo	110/196 (56.1%)	49.2–63%	61/196 (31.1%)	24.6–37.6%	37/196 (18.8%)	13.5–24.3%
Breed	*p* = 0.338		*p* = 0.530		*p* = 0.457	
Mixed-breed	549/1113 (49.3%)	46.4–52.3%	270/1113 (24.2%)	21.7–26.8%	158/1113 (14.2%)	12.1–16.2%
Pure-breed	79/174 (45.4%)	38–52.8%	46/174 (26.4%)	19.8–33%	21/174 (12.1%)	7.2–16.9%

95% confidence interval (CI); * *p* <0.05.

## Data Availability

Data are contained within the article.
